# Lung tumor motion reproducibility for five patients who received four-fraction VMAT stereotactic ablative body radiotherapy under constrained breathing conditions: a preliminary study

**DOI:** 10.1093/jrr/rru055

**Published:** 2014-06-25

**Authors:** Keiichi Nakagawa, Akihiro Haga, Katsutake Sasaki, Satoshi Kida, Yoshitaka Masutani, Hideomi Yamashita, Wataru Takahashi, Hiroshi Igaki, Akira Sakumi, Kuni Ohtomo, Kiyoshi Yoda

**Affiliations:** 1 University of Tokyo Hospital, Department of Radiology, Hongo, Bunkyo-ku, Tokyo, Japan; 2 Elekta KK, Research Physics, Shibaura, Minato-ku, Tokyo, Japan

**Keywords:** tumor motion reproducibility, VMAT, SABR

Dear Editor,

Several different approaches have been applied to stereotactic hypofractionated radiotherapy for lung tumors, including free breathing, breath-hold, gating, and tracking. Negoro *et al.* reported that abdominal compression reduced the movement of lung tumors (thereby possibly reducing treatment uncertainty), with portal fluoroscopy being used to measure the tumor movement [1]. Heinzerling *et al.* [2] and Han *et al.* [3] confirmed the validity of abdominal compression using 4D computed tomography (CT). Bouilhol *et al.* also reconfirmed the validity of abdominal compression using 4D CT and reported that the internal target volume was significantly reduced for lower lobe tumors [4]. On the other hand, Bissonnette *et al.* reported that abdominal compression increased the variation of tumor motion by referring to their 4D cone-beam CT (CBCT) data, contending that longer treatment time to include the abdominal compression procedure may reduce the reproducibility of tumor motion [5]. Richmond *et al.* [6] and Mampuya *et al*. [7] also reported significant variation in the average tumor position under abdominal compression from their 3D CBCT data.

Using a 4D planning CT imager, Aquilion LB, (Toshiba, Ohtawara, Japan) we calculated 3D lung tumor motion trajectories with an Anzai belt (Anzai, Tokyo, Japan) and stereotactic body frame with an abdominal compression plate (Elekta AB, Stockholm, Sweden) for five patients who received four-fraction VMAT stereotactic ablative body radiotherapy (SABR). In addition, the motion trajectories of lung tumors were calculated using 4D CBCT imaging functionality provided by an X-ray Volume Imaging (XVI) system version 4.5 (Elekta AB, Stockholm, Sweden) both immediately before and during treatment. The pre-treatment 4D CBCT data were acquired by the built-in XVI software, Symmetry, whereas in-treatment 4D CBCT was obtained by in-house software using projection images acquired during VMAT delivery [8]. The breathing amplitudes obtained using the 4D planning CT and 4D CBCT were divided into five equal intervals, i.e. ten breathing phases. Subsequently, the trajectory was obtained by calculating each gravity center of the tumor for each phase. The resulting trajectory was visually inspected to analyze the reproducibility of the tumor motion at the time of planning, immediately before treatment and during treatment. The data acquisition times for pre-treatment and in-treatment CBCT are typically 4 min and 3.5 min, respectively. Therefore, the calculated trajectories are time-averaged during these periods. As reported previously, a large variation in the average tumor position was observed between planning and pre-treatment CBCT imaging. However, this offset would be automatically corrected by the XVI software, Symmetry, after automatic bone matching, so that the patient couch would be repositioned according to a time-averaged tumor position on each treatment day. Having this clinical workflow in mind, tumor motion reproducibility was analyzed after subtracting the average 3D position from each trajectory.

**Fig. 1.**
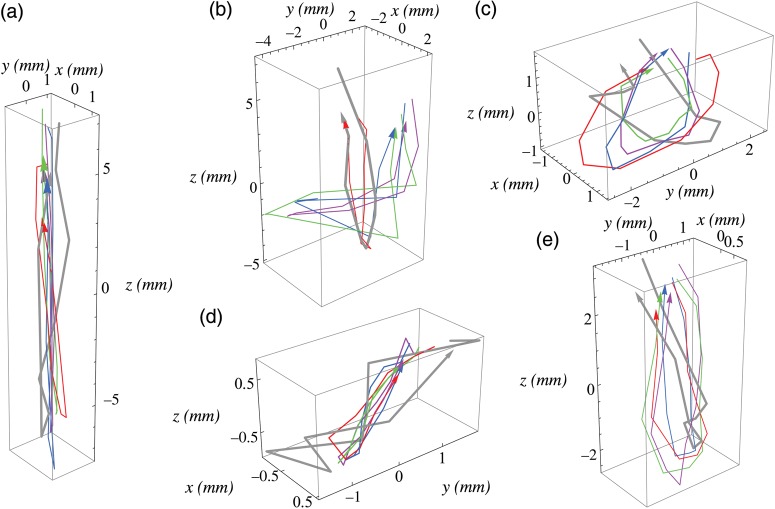
3D lung tumor trajectories during the planning time (in gray) and pre-treatment times in the four fractions (in red, green, blue and violet) for the five patients. The *x*, *y* and *z* axes correspond to the lateral, anteroposterior and craniocaudal directions, respectively.

Figure 1a–e shows the lung tumor trajectories during the planning times (in gray) and pre-treatment times in the four fractions (in red, green, blue and violet) for the five patients. Throughout this letter, the *x*, *y* and *z* axes correspond to the lateral, anteroposterior and craniocaudal directions, respectively. A large interpatient variability was observed: Fig. 1a shows nearly one-dimensional movement in the craniocaudal direction. Figure 1c–e shows much smaller but more isotropic tumor movements with significant hysteresis. This may be due to variation in the tumor locations, the abdominal compression forces, and the compressed positions between patients. In addition, if we consider a typical lung tumor having a dimension of 10 mm or larger, the trajectory differences between the planning and pre-treatment times for each patient may be clinically ignored. Figure 1a–e also suggests that 4D CBCT may be used for calculating the internal target volume (ITV) and the planning target volume (PTV).

**Fig. 2.**
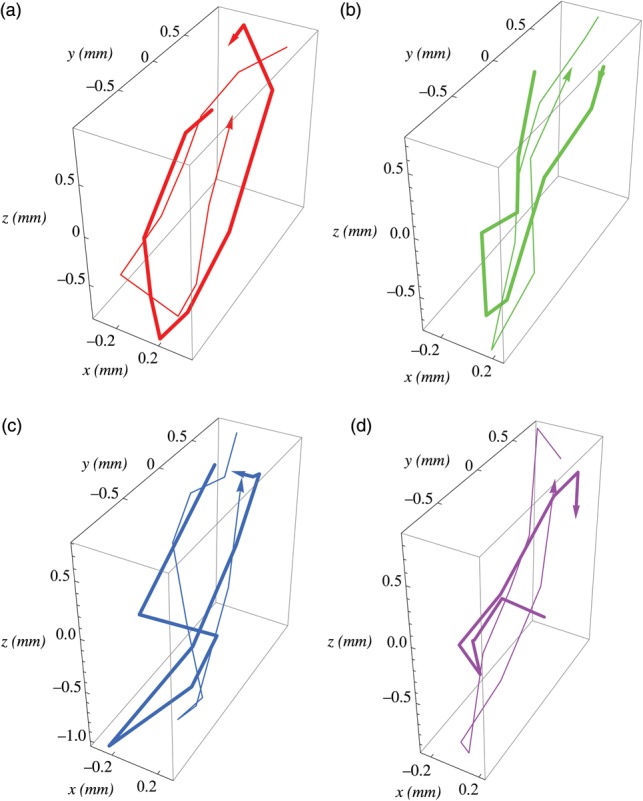
3D lung tumor trajectories obtained by pre-treatment 4D CBCT (thin line) and those obtained by in-treatment 4D CBCT (thick line), fraction by fraction, for a patient. The *x*, *y* and *z* axes correspond to the lateral, anteroposterior and craniocaudal directions, respectively.

**Fig. 3.**
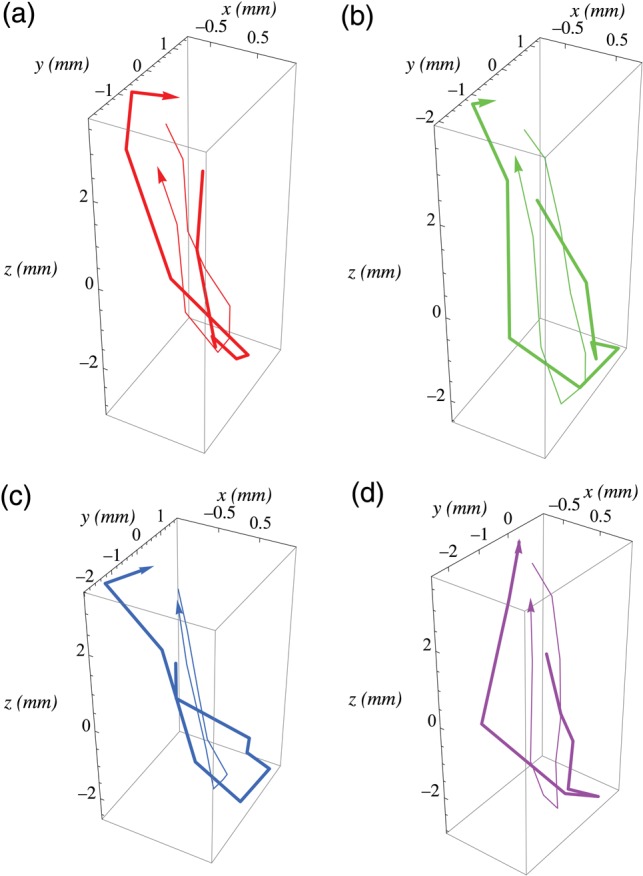
3D lung tumor trajectories obtained by pre-treatment 4D CBCT (thin line) and those obtained by in-treatment 4D CBCT (thick line), fraction by fraction, for a different patient. The *x*, *y* and *z* axes correspond to the lateral, anteroposterior and craniocaudal directions, respectively.

Figure 2a–d compares lung tumor trajectories obtained by pre-treatment 4D CBCT (thin line) with those obtained by in-treatment 4D CBCT (thick line), fraction by fraction, for a patient. Figure 3a–d shows another trajectory comparison for a different patient. Again, if we consider a tumor size of 10 mm or larger, the observed differences between pre-treatment and in-treatment times may be clinically ignored.

In conclusion, we confirmed the reproducibility of lung tumor movement using 4D planning CT and 4D CBCT for five patients who received four-fraction VMAT SABR under constrained breathing conditions. The results appear to be clinically acceptable, but further study is needed because of the small data size of this preliminary study. It is anticipated that the flattening-filter-free technique may increase breathing trajectory reproducibility due to its faster dose delivery [9]. In addition, reproducibility should also be discussed in terms of dose calculation in 4D [10]. The current study is in compliance with the ethical guidelines of the hospital, and written informed consent was obtained before the treatment was initiated.
